# HOXC8 promotes proliferation and migration through transcriptional up-regulation of TGFβ1 in non-small cell lung cancer

**DOI:** 10.1038/s41389-017-0016-4

**Published:** 2018-01-17

**Authors:** Houli Liu, Mingsheng Zhang, Shanshan Xu, Jie Zhang, Jin Zou, Chenchen Yang, Yang Zhang, Chen Gong, Yuanzhong Kai, Yong Li

**Affiliations:** 1 School of Life Sciences, Anhui University, Hefei, Anhui Province China; 2 Department of Oncology, Tongji Hospital, Tongji Medical College, Huazhong University of Science and Technology, Wuhan, Hubei China

## Abstract

Homeobox (HOX) genes encode a family of transcription factors, which play crucial roles in numerous processes, and their dysregulation is involved in the carcinogenesis of many human cancers. In the present study, we investigated the roles of HOXC8 in non-small cell lung cancer (NSCLC). We showed that HOXC8 was upregulated in clinical NSCLC specimens compared to normal lung tissues, and the high expression of HOXC8 correlated with tumor node metastasis (TNM) stage, tumor status, lymph nodal status and poor relapse-free survival for lung cancer patients. Functionally, HOXC8 expression significantly promoted the proliferation, anchorage-independent growth and migration of NSCLC, and HOXC8 functioned as a transcription activator to induce the expression of TGFβ1, leading to an increase in the proliferation, anchorage-independent growth and migration of NSCLC. Furthermore, we demonstrated that HOXC8 expression was associated with chemoresistance and anti-apoptosis in NSCLC, suggesting that HOXC8 is a promising therapeutic target for chemosensitization of NSCLC to cisplatin. Altogether, our study defined a critical role of HOXC8 in promoting transcription of TGFβ1 and NSCLC tumorigenesis.

## Introduction

Lung cancer is the leading cause of cancer deaths, accounting for more than one-quarter of cancer-related deaths worldwide^1^. Non-small cell lung cancer (NSCLC), which includes lung adenocarcinoma (LUAD), lung squamous cell carcinoma (LUSC), and large cell carcinoma, accounts for >80% of all lung cancers^2^. Currently, the incidence of NSCLC is increasing; however, the survival rates for NSCLC patients remain poor, mostly owing to migration and metastasis of lung cancer cells^3^. Therefore, it is important to identify the molecular mechanisms underlined in the regulation of lung cancer cell aggressiveness.

The homeobox (HOX) genes constitute a family of transcription factors that participate in a number of physiological processes, including embryonic development, cell proliferation and differentiation, etc^4^. Numerous evidences show that HOX genes are deregulated in multiple cancers such as prostate cancer, pancreatic cancer, breast cancer and lung cancer, in which deregulation of HOX genes can promote or repress cancer processes^5–8^. For example, overexpression of HOXA9 is associated with high-risk acute myeloid leukemia by participating the regulation of Syk activity^9^. HOXB7 expression is significantly upregulated in colorectal cancer, and expression of HOXB7 promotes the aggressiveness of cancer cells^10^. HOXD8 was found to be downregulated in colorectal cancer and functioned as a tumor suppressor in colorectal cancer progression^11^. In our previous studies, we reported that HOXC8 was upregulated in breast cancer cells and ectopic expression of HOXC8 promoted breast cancer migration and metastasis^8,12,13^. Moreover, HOXC8 was found to be upregulated in cervical cancer, prostate cancer and colorectal cancer and facilitated the migration and metastasis of cancer cells^5,14–16^.

Transforming growth factor beta 1 (TGFβ1) is an important cytokine in cancer progression, and plays a very important role in migration and metastasis of various cancers^17–19^. In this study, we investigated HOXC8 expression in NSCLC clinical specimens and normal tissues by immunohistochemistry. We further explored the effects of HOXC8 on NSCLC cell proliferation, anchorage-independent cell growth and migration by ectopically expressing or shRNA silencing HOXC8 expression in NSCLC cells. We found that HOXC8 contributed to NSCLC cell proliferation, anchorage-independent and migration via regulating TGF-β1 expression, and high expression of HOXC8 was associated with aggressive phenotypes and poor relapse free survival for lung cancer patients. This study may provide a novel molecular mechanism underlying NSCLC proliferation and migration, and help for developing new therapeutic strategies for NSCLC.

## Results

### HOXC8 expression is up-regulated in NSCLC

**Fig. 1:**
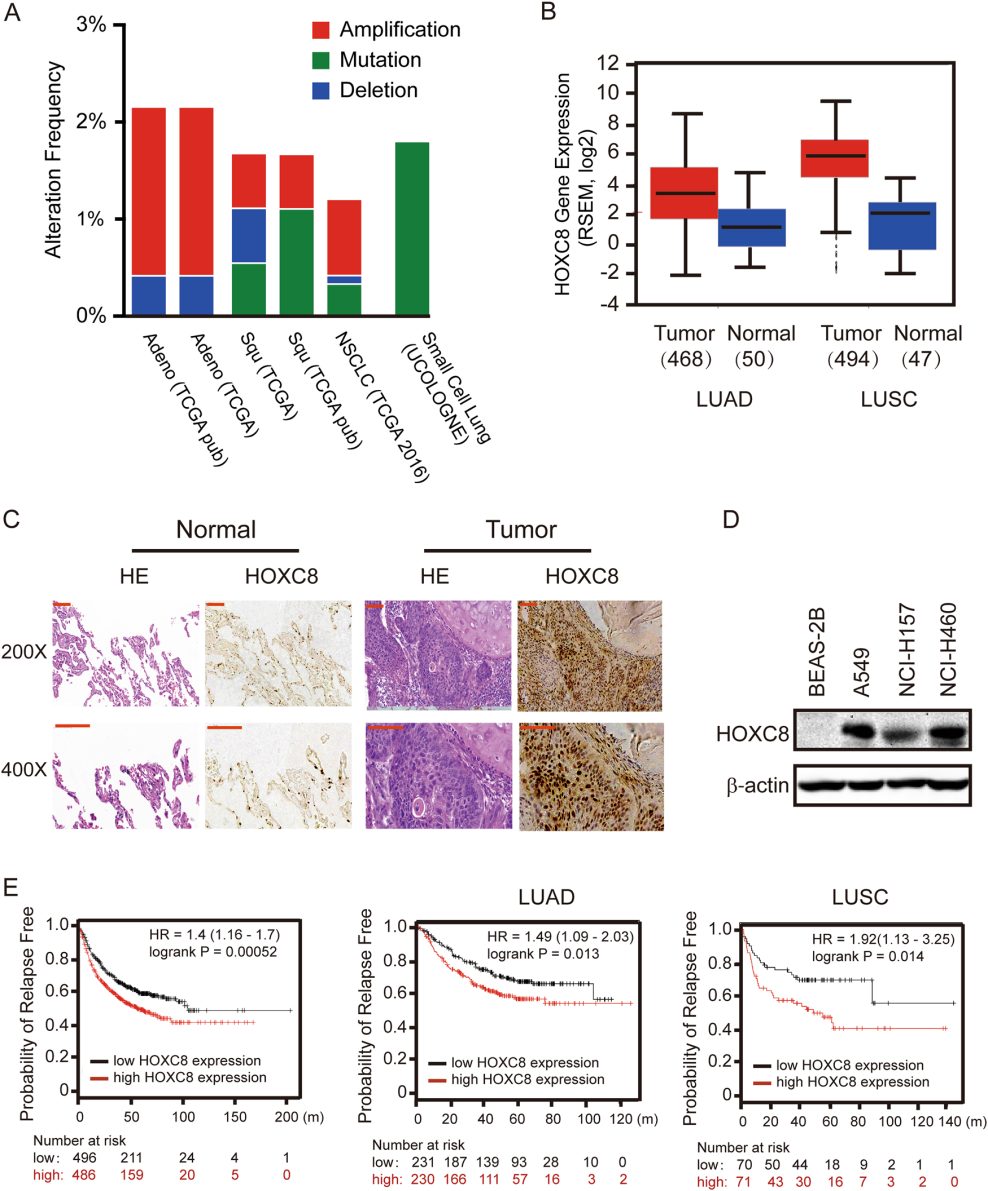
HOXC8 expression is elevated in lung cancer. **a** Alteration frequency of HOXC8 in multiple lung cancer types. Data were obtained from The Cancer Genome Atlas portal: http://www.cbioportal.org **b** HOXC8 mRNA levels between lung clinical specimens and normal samples of LUAD or LUSC using publicly available data sets (FIREHOSE broad GADC, http://gdac.broadinstitute.org). **c** Representative immunohistochemical images of HOXC8 expression in clinical lung cancer specimens and normal lung tissues (200×, upper panel; 400×, lower panel). Scale bar=100 μm. **d** Western blot showing the protein levels of HOXC8 in lung cancer cell lines (A549, NCI-H157 and NCI-H460) and normal lung cell line (BEAS-2B). β-actin was used as the loading control. **e** Kaplan–Meier survival plots show relapse-free survival for lung cancer patients with high and low expression of HOXC8. (http://kmplot.com, probe ID: 221350_at for HOXC8). KM plots for whole lung cancer patients (left), LUAD patients (middle), and LUSC patients (right)

Previous studies indicate that HOXC8 plays an important role in multiple cancer progression, however, little is known about HOXC8 roles in lung cancer^13,20–23^. To explore the functions of HOXC8 in lung cancer development, we first examined the alteration frequency of HOXC8 in lung cancer using the publicly available data sets (cBioportal, www.cbioportal.org). We found that HOXC8 showed high alteration frequency in NSCLC, particularly in adenocarcinoma, in comparison with small lung cancer (Fig. 1a). Moreover, bioinformatics analysis using the data from Broad GADC Firehose (http://gdac.broadinstitute.org) indicated that LUAD and LUSC exhibited higher HOXC8 mRNA levels in clinical cancer specimens than in normal samples (Fig. 1b). To provide further evidence, we performed immunohistochemistry (IHC) to examine HOXC8 expression levels in NSCLC specimens. The results showed that HOXC8 was lowly expressed in normal lung tissues, meanwhile, it was markedly elevated and located mainly in cell nucleus in NSCLC clinical specimens (Fig. 1c; Supplementary Fig S1). The high HOXC8 expression rate (78.9%) was significantly higher in cancer specimens compared to normal lung tissues (16.7%) (Table 1). Notably, the expression difference between cancer specimens and normal tissues are statistically significant for HOXC8 (*P* < 0.0001) (Table 1). By western blot, we found that HOXC8 expression was upregulated in NSCLC cell lines (A549, NCI-H157 and NCI-H460) compared to normal human bronchial epithelial cell line BEAS-2B (Fig. 1d).

**Table 1:** IHC staining of HOXC8 in clinical NSCLC specimens and normal lung tissues

	Low expression	High expression	*P*-value
Tumor tissues (*n* = 38)	8 (21.1%)	30 (78.9%)	<**0.0001**
Normal tissues (*n* = 18)	15 (83.3%)	3 (16.7%)	

The bold values indicate that the differences are statistically significant as the *P-*value < 0.05

We further analyzed the correlation between HOXC8 expression and the clinical pathological factors in NSCLC tissues. As summarized in Table 2, HOXC8 expression had significantly positive correlation with TNM stage (*P* = 0.0378), higher tumor size (*P* = 0.0212) and positive nodal status (*P* = 0.0217), suggesting an important association between HOXC8 upregulation and tumor proliferation and metastasis. Moreover, online Kaplan–Meier survival analysis (http://kmplot.com) showed that patients with higher expression of HOXC8 had significantly lower probability of relapse free survival for lung cancer patients (logrank *P* = 0.00052), as well as LUAD patients (logrank *P* = 0.013) and LUSC patients (logrank *P* = 0.014) (Fig. 1e). Taken together, all the results above strongly suggested that HOXC8 played an important role in the development and progression of NSCLC.

**Table 2:** Correlation of HOXC8 expression with clinicopathological characteristics in NSCLC

Characteristics	Number	Low expression	High expression	*P-*value
Age				
<60	16	4	12	0.825
≥60	22	4	18	
Gender				
Male	18	3	15	0.399
Female	20	5	15	
Histology				
Adenocarcinoma	23	4	19	0.541
Squamous cell carcinoma	15	4	11	
TNM stage				
I II	25	7	18	**0.0378**
III IV	13	1	12	
Tumor status				
T1	15	6	9	**0.0212**
T2 T3 T4	23	2	21	
Nodal status				
N0	24	7	17	**0.0217**
N1 N2 N3	14	1	13	

The bold values indicate that the differences are statistically significant as the *P-*value < 0.05

### HOXC8 is required for lung cancer cell growth and migration

**Fig. 2:**
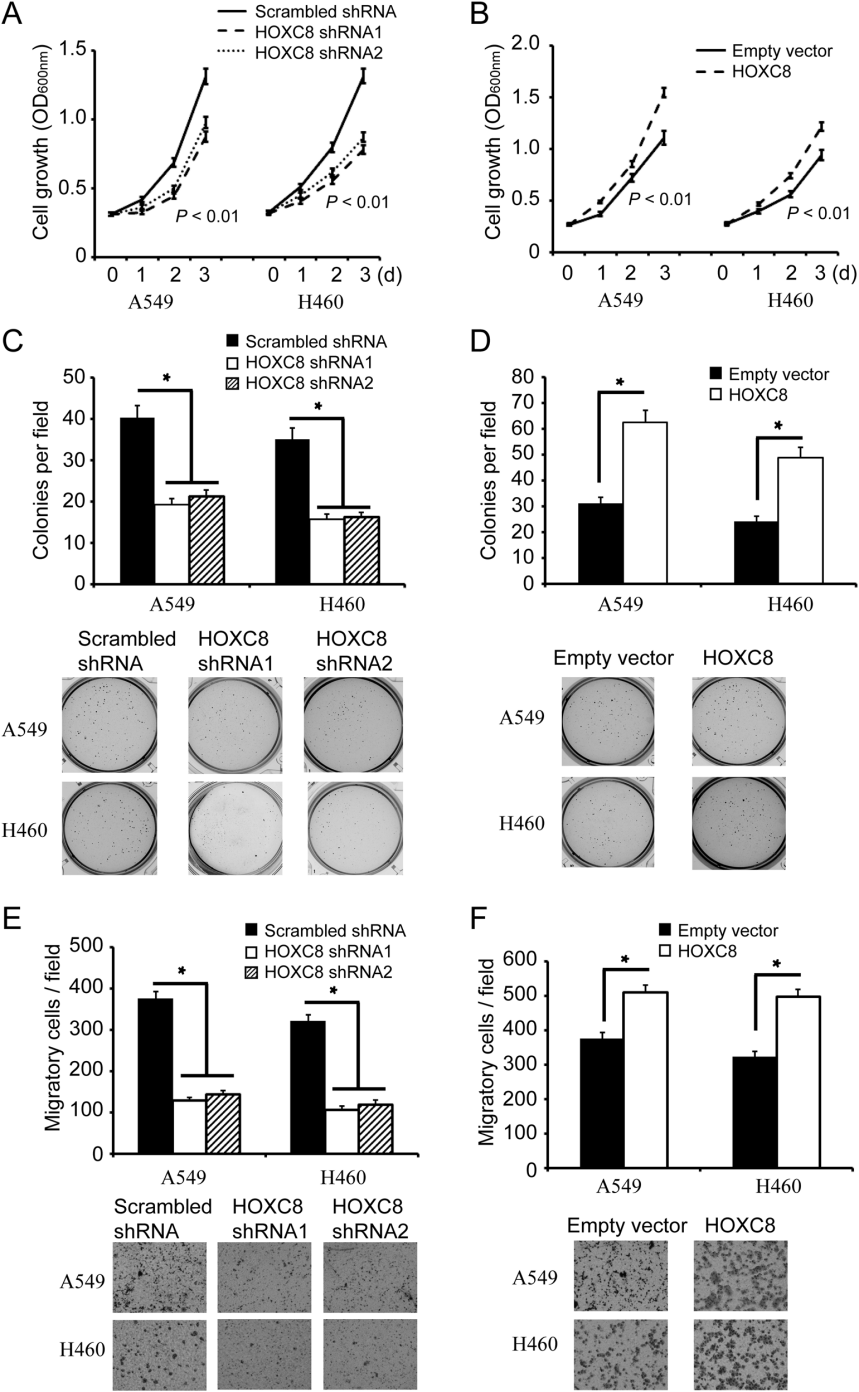
HOXC8 promotes the proliferation, anchorage-independent growth and migration of NSCLC cells. **a** MTT assays to analyze the proliferation of control or HOXC8 shRNA knockdown cells. Data are the mean ± SEM; *n* = 3; *P* < 0.01. **b** MTT assays were carried out in A549 or H460 cells that were lentivirally transduced with vectors encoding HOXC8 or empty vectors as control. Data are the mean ± SEM; *n* = 3; *P* < 0.01. **c** Control (scrambled sequence) or HOXC8-knockdown cells were subjected to soft agarose colony formation. Columns, mean; bars, SEM; n = 3; *P < 0.05 **d** Cells transduced with HOXC8 expression vectors were subjected to soft agarose colony formation. Columns, mean; bars, SEM; *n* = 3; *P < 0.05. **e** Transwell assays to analyze cell migration of control or HOXC8 shRNA knockdown cells. Columns, mean; bars, SEM; *n* = 3; * *P* < 0.05. **f** Transwell assays to analyze migration of cells transduced with empty vectors or HOXC8 expression vectors. Columns, mean; bars, *n* = 3; SEM; * *P* < 0.05

To determine the role of HOXC8 in lung cancer cells, we carried out experiments to knockdown HOXC8 expression or ectopically express HOXC8 in NSCLC cell line A549 or NCI-H460. HOXC8 shRNAs knockdown led to a clear reduction in HOXC8 expression at both protein levels and mRNA levels, and HOXC8 ecto-expression greatly increased both HOXC8 protein levels and mRNA levels in A549 or NCI-H460 cells (Supplementary Fig. S2). In MTT assays, silencing HOXC8 significantly reduced lung cancer cell proliferation as shown by the growth curves (Fig. 2a), and ectopic expression of HOXC8 markedly increased the proliferation of A549 or NCI-H460 (Fig. 2b).

Next, we tested whether HOXC8 was required for anchorage-independent growth by using soft-agar assays. We found that HOXC8 shRNA knockdown in A549 or NCI-H460 cells significantly inhibited the colony formation of the cancer cells (Fig. 2c), and ectopic expression of HOXC8 enhanced the colony formation of cancer cells (Fig. 2d). Finally, to investigate the role of HOXC8 in migration of lung cancer cells, we performed transwell migration assays. We found that HOXC8 shRNA knockdown resulted in significant reduction of migratory cells (Fig. 2e), and ecto-expression of HOXC8 significantly increased the migration of cancer cells (Fig. 2f). Collectively, the above data indicated that HOXC8 played an essential role in growth and migration of NSCLC cells.

### HOXC8 regulates the expression of EMT-related genes

Epithelial-to-mesenchymal transition (EMT) is a crucial event for migration and metastasis of various cancers^24^. EMT is a characterized by loss of epithelial markers and upregulation of mesenchymal markers, leading to the transition from epithelial phenotype to mesenchymal fibroblast-like cells^25,26^. We observed that some of A549 cells with ectopic HOXC8 expression underwent EMT-like elongation to become fibroblast-like spindle-shaped cells (data not shown), so we performed qRT-PCR experiments to examine the expression changes of EMT-related genes in A549 cells transduced with HOXC8 ecto-expression vectors or HOXC8 shRNA vectors. Among those EMT-related genes, we found that HOXC8 is probably involved in regulating the expression of genes such as E-cadherin, vimentin and TGFβ1 in A549 cells (Supplementary Fig. S3).

**Fig. 3:**
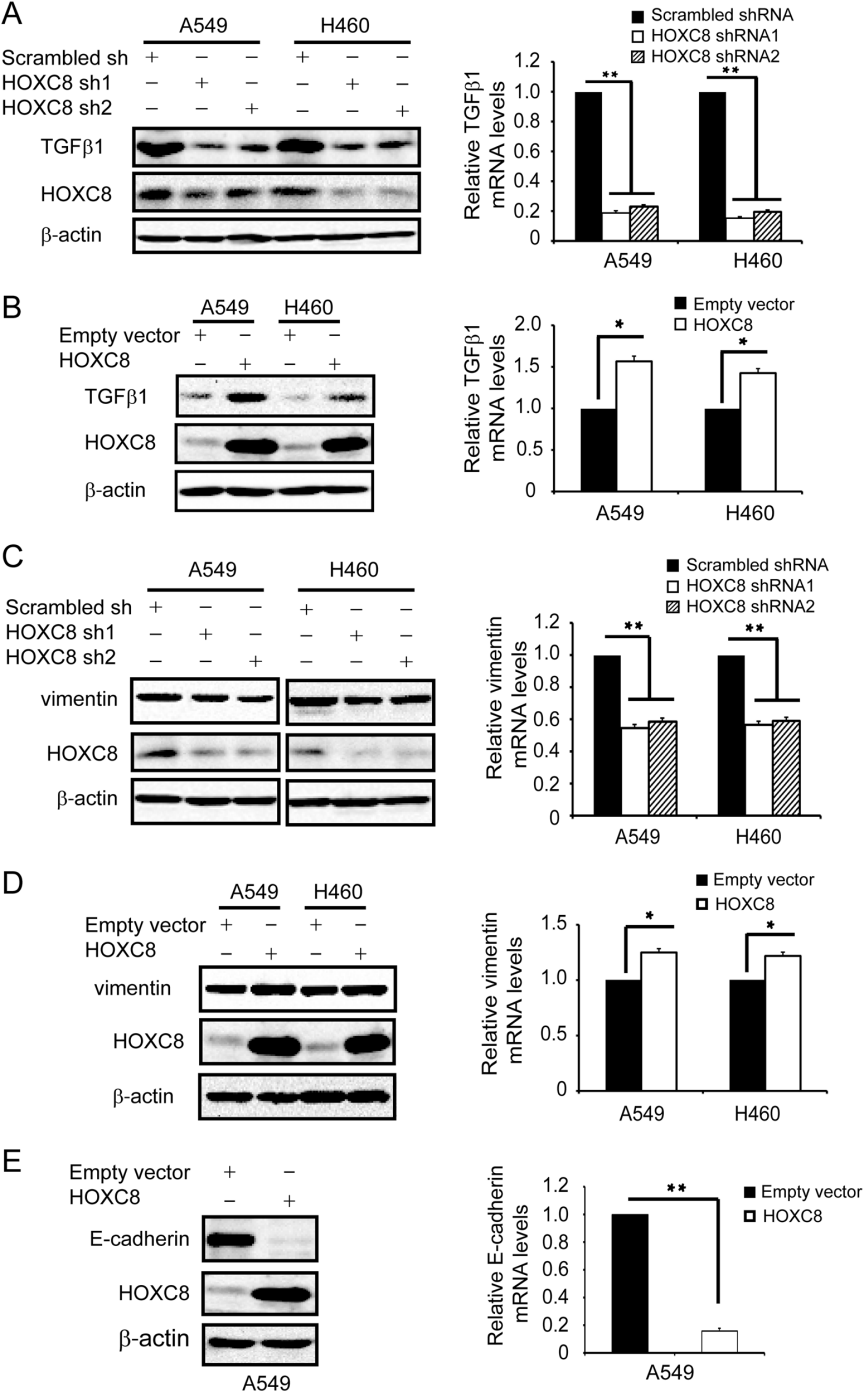
HOXC8 regulates the expression of EMT-associated genes. **a** Cells were lentivirally transduced with scrambled or HOXC8 shRNAs. Cell lysates were subjected to Western blot to detect TGFβ1, HOXC8, and β-actin (left panel). Total RNA was subjected to qRT-PCR to measure the levels of TGFβ1 mRNA (right panel); β-actin mRNA was used as an internal control for standardization. Columns, means; bars, SEM; *n* = 3; **P < 0.01. **b** Cells were lentivirally transduced with empty vectors or HOXC8 expression vectors. Cell lysates were subjected to Western blot to detect TGFβ1, HOXC8, and β-actin (left panel). Total RNA was subjected to qRT-PCR to measure the level of TGFβ1 mRNA (right panel); β-actin mRNA was used as an internal control for standardization. Columns, means; bars, SEM; *n* = 3; **P < 0.01. **c** Western blot was performed to examine vimentin protein levels in cells that lentivirally transduced with scrambled or HOXC8 shRNAs (left panel). qRT-PCR was used to examine vimentin mRNA levels in HOXC8 shRNA knockdown cells (right panel), β-actin was used as an internal control for standardization. Columns, means; bars, SEM; *n* = 3; **P < 0.01. **d** Western blot was performed to examine vimentin protein levels in cells that lentivirally transduced with empty vectors or HOXC8 expression vectors (left panel). qRT-PCR was used to examine vimentin mRNA levels in cells that lentivirally transduced with empty vectors or HOXC8 expression vectors (right panel), β-actin was used as an internal control for standardization. Columns, means; bars, SEM; *n* = 3; *P < 0.05. **e** Western blot was performed to examine E-cadherin protein levels in A549 cells transduced with HOXC8 expression vectors (left panel). qRT-PCR was used to examine E-cadherin mRNA levels in cells transduced with HOXC8 expression vectors (right panel), β-actin was used as an internal control for standardization. Columns, means; bars, SEM; *n* = 3; *P < 0.05. NCI-H460 cells were not included as they did not express E-cadherin protein

To confirm whether HOXC8 regulates these genes expression, we further carried out western blot and qRT-PCR experiments in A549 or NCI-H460 cells that were lentivirally transduced with HOXC8 shRNAs vectors or HOXC8 expression vectors. As expected, depletion of HOXC8 resulted in significant reduction in the TGFβ1 protein and mRNA levels in A549 and NCI-H460 cells (Fig. 3a), and ectopic expression of HOXC8 obviously increased the protein and mRNA levels of TGFβ1 in both A549 and NCI-H460 cells (Fig. 3b). Moreover, silencing HOXC8 decreased the protein and mRNA levels of vimentin (Fig. 3c), and forced expression of HOXC8 increased vimentin protein and mRNA levels in both A549 and NCI-H460 cells (Fig. 3d). In addition, ectopically expressing HOXC8 in A549 cells led to significant reduction in E-cadherin protein or mRNA levels (no E-cadherin expression was detected in NCI-H460 cells) (Fig. 3e). Taken together, these data indicated that HOXC8 was involved in regulating TGFβ1, vimentin and E-cadherin genes expression in NSCLC cells.

### HOXC8 regulates TGFβ1 transcription

To test whether HOXC8 directly regulates these genes transcription, we carried out chromatin immunopreciption (ChIP) to investigate whether HOXC8 binds to the promoter of these genes, and we found that HOXC8 bound to the promoter of TGFβ1 in A549 and NCI-H460 cells.

**Fig. 4:**
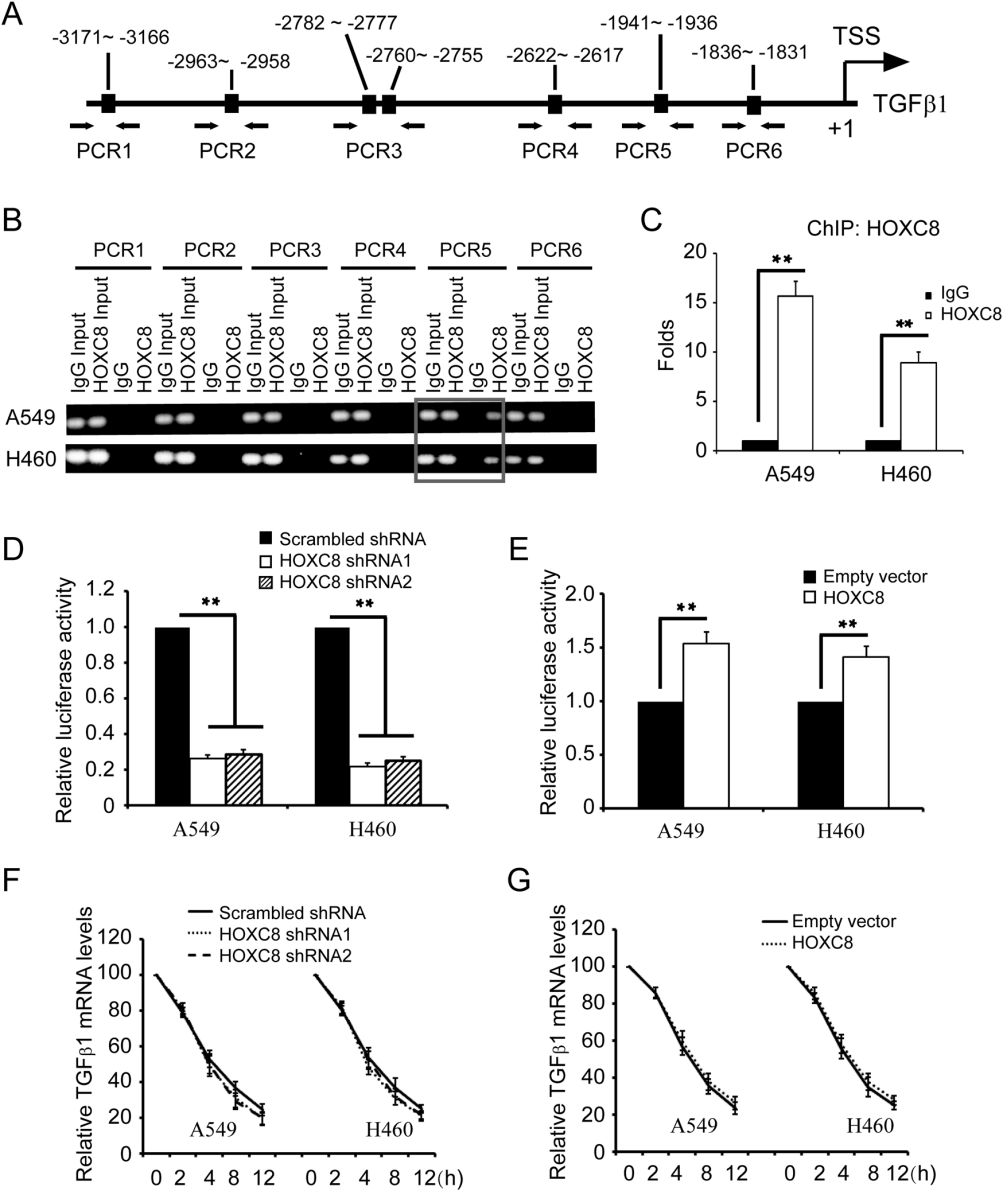
HOXC8 functions as TGFβ1 transcription factor in NSCLC cells. **a** Positions of PCR primers designed to amplify the regions containing putative HOX binding sites in TGFβ1 promoter. **b**, **c** ChIP was performed using the HOXC8 antibody, and the immunoprecipitated chromatin DNA was subjected to PCR (**b**) or qPCR (**c**). Each sample was run in triplicate and in multiple experiments for mean ± SEM; **P < 0.01. **d** Luciferase assays were performed in cells transduced with TGFβ1 promoter luciferase reporter vectors in HOXC8 shRNA knockdown cells. The luciferase activity was normalized to the Renilla activity. Columns, means; bars, SEM; *n* = 3; **P < 0.01. **e** Luciferase assays were performed in cells transduced with TGFβ1 promoter luciferase reporter vectors in HOXC8 ecto-expression cells. Columns, means; bars, SEM; n = 3; *P < 0.05. **f**, **g** HOXC8 knockdown cells or HOXC8 ectopically expressing cells were transfected with 2 μg/ml actinomycin for indicated time. Total RNA was isolated and subjected to qRT-PCR to measure the level of TGFβ1 mRNA. GAPDH mRNA was used as an internal control. The level of TGFβ1 mRNA without actinomycin treatment was considered as 100%. Values are means ± SEM; *n* = 3

Based on reported HOX protein binding sequences^27^, we analyzed the sequence of TGFβ1 promoter and found several putative HOX protein binding sites in TGFβ1 promoter. We designed several sets of PCR primers that specifically amplified each region containing the putative HOX binding sequence in TGFβ1 promoter (Fig. 4a) and performed ChIP using anti-HOXC8 antibody and IgG as negative control. ChIP analyses showed that HOXC8 bound to TGFβ1 promoter on nucleotides −1941 to −1936 (Fig. 4b), and quantitative PCR further showed that the region was enriched 8–16 folds in the HOXC8 chromatin immunoprecipitates compared to IgG negative control in A549 or NCI-H460 cells (Fig. 4c). These data indicated that HOXC8 bound to the nucleotides −1941 to −1936 in TGFβ1 promoter in NSCLC cells.

To further determine whether HOXC8 functions as a transcription factor to regulate TGFβ1 transcription, we subcloned TGFβ1 promoter region into firefly luciferase reporter vector pGL4.23. Luciferase analyses showed that silencing HOXC8 resulted in significant decreases in the luciferase activities (Fig. 4d), and expressing HOXC8 greatly enhanced the luciferase activities compared to the negative control (Fig. 4e). Next, we performed deletion mutagenesis to delete the HOXC8 binding site in TGFβ1 promoter, and found that the mutation completely abolished HOXC8 effects on the activities of TGFβ1 promoter (Supplementary Fig. S4). Moreover, actinomycin-chasing experiments further demonstrated that HOXC8 shRNA knockdown or ectopic expression did not affect TGFβ1 mRNA stability (Fig. 4f, g). Collectively, the above data indicated that HOXC8 functioned as a transcription factor to activate TGFβ1 transcription in NSCLC cells.

### HOXC8-TGFβ1 pathway is involved in proliferation, anchorage-independent cell growth and migration of NSCLC cells

**Fig. 5:**
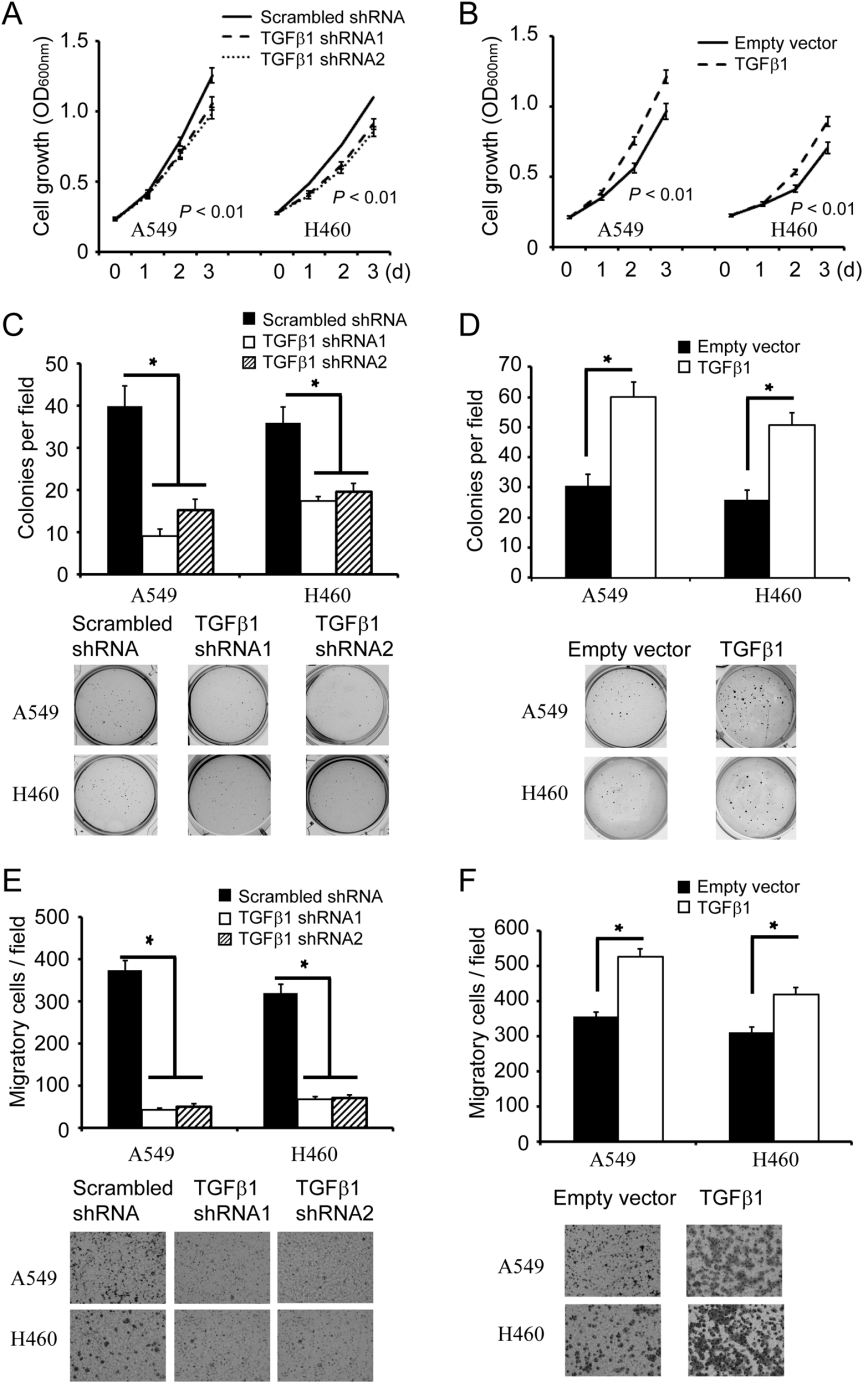
TGFβ1 promotes the proliferation, anchorage-independent growth and migration of NSCLC cells. **a** MTT assays to analyze the proliferation of control or HOXC8 shRNA knockdown cells. Data are the mean ± SEM; *n* = 3; *P* < 0.01. **b** MTT assays were carried out in cells transduced with vectors encoding HOXC8 or empty vectors as control. Data are the mean ± SEM; *n* = 3; *P* < 0.01. **c** Soft agar assays for anchorage-independent growth were carried out in cells transduced with scrambled or TGFβ1 shRNAs. Columns, mean; bars, SEM; *n* = 3; *P < 0.05 **d** Soft agar assays for anchorage-independent growth were carried out in cells transduced with HOXC8 expression vectors or empty vectors. Columns, mean; bars, SEM; *n* = 3; *P < 0.05. **e** Transwell assays to analyze the migration of control or TGFβ1 shRNA knockdown cells. Columns, mean; bars, SEM; *n* = 3; *P < 0.05. **f** Transwell assays to analyze the migration of cells transduced with TGFβ1 expression vectors or empty vectors. Columns, mean; bars, *n* = 3; SEM; * *P* < 0.05

Given the fact that HOXC8 regulates TGFβ1 transcription in lung cancer cells, we analyzed the effects of TGFβ1 on cell proliferation, anchorage-independent cell growth and cell migration in A549 and NCI-H460 cells. MTT assays showed that knockdown of TGFβ1 significantly decreased the proliferation of A549 or NCI-H460 cells, and ectopic expression of TGFβ1 markedly increased the cell proliferation (Fig. 5a, b). In soft-agar colony formation assay, depletion of TGFβ1 significantly impaired anchorage-independent cell growth of both A549 and NCI-H460 cells (Fig. 5c), and ectopic expression of TGFβ1 greatly increase anchorage-independent cell growth (Fig. 5d). Transwell assays further showed that knockdown of TGFβ1 significantly inhibited the migration of A549 or NCI-H460 cells (Fig. 5e), and TGFβ1 ecto-expression enhanced the migration of lung cancer cells (Fig. 5f). These observations suggested that TGFβ1 participated in the proliferation, anchorage-independent cell growth and migration of NSCLC cells.

**Fig. 6:**
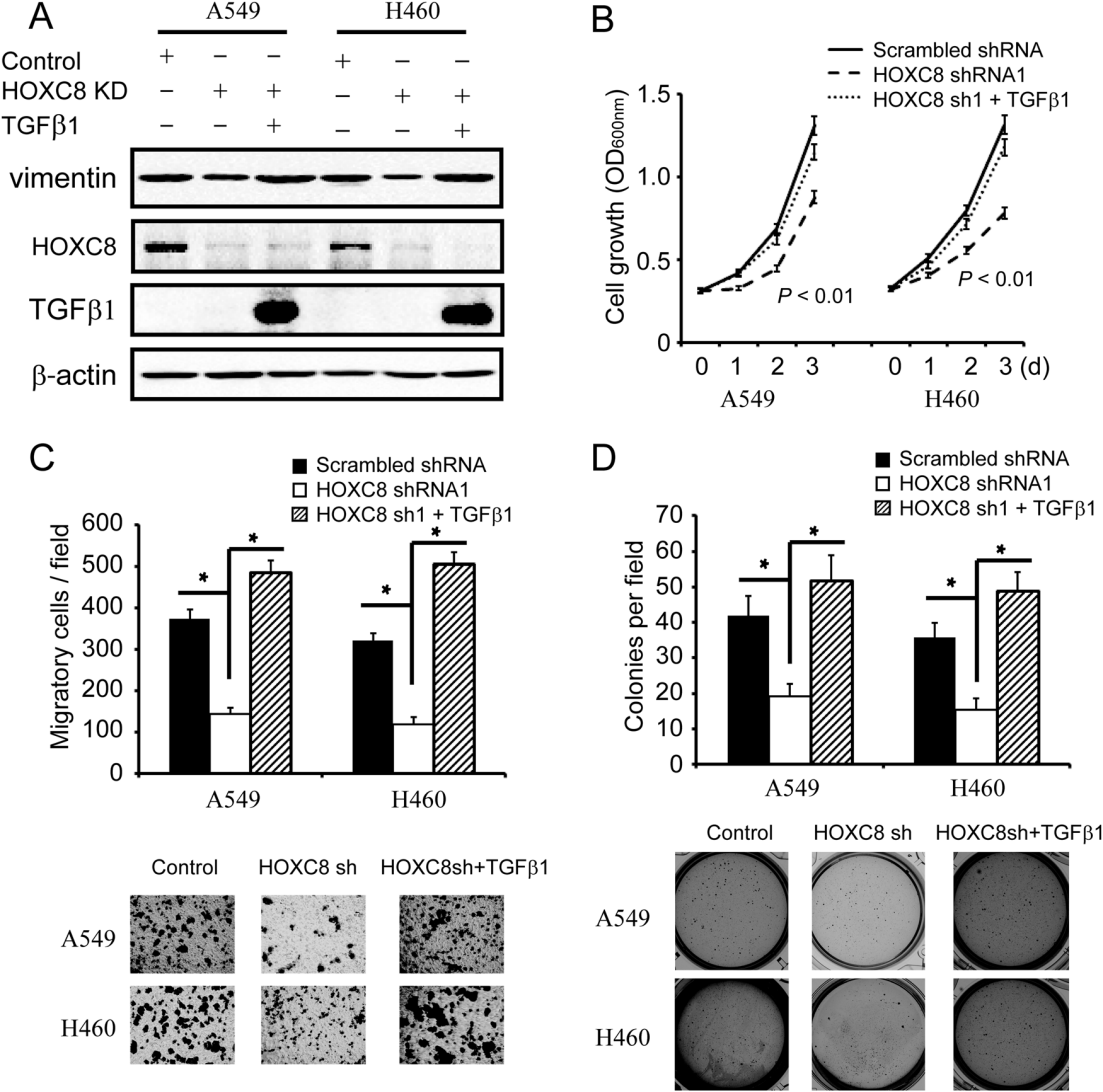
HOXC8-TGFβ1 pathway is required for the proliferation, anchorage-independent cell growth and migration of NSCLC cells. A549 or NCI-H460 cells were lentivirally transduced with scrambled or HOXC8 shRNA vectors, and then transduced with TGFβ1 expression vectors. **a** Cell lysates were subjected to Western blot to detect TGFβ1, HOXC8, and β-actin. **b** MTT assays were performed to examine cell proliferation. Data are the mean ± SEM; *n* = 3; *P* < 0.01. **c** Soft agar assays were carried out to examine the anchorage-independent growth. Columns, mean; bars, SEM; *n* = 3; * *P* < 0.05. **d** Transwell assays were performed to analyze cell migration. Columns, means; bars, SEM; *n* = 3; *P < 0.05

To explore whether the effects of HOXC8 were functionally linked to its regulation of TGFβ1 transcription, HOXC8 knockdown cells were lentivirally transduced with TGFβ1 expression vectors. In both A549 and NCI-H460, HOXC8 silencing by shRNA knockdown decreased the expression of vimentin, which was completely recovered by ectopic expression of TGFβ1 in HOXC8 knockdown cells (Fig. 6a). Moreover, HOXC8 knockdown cells displayed an impaired capability for proliferation (Fig. 6b), colony formation (Fig. 6c) and migration (Fig. 6d), which was almost restored by ectopically expressing TGFβ1 in the HOXC8-knockdown cells (Fig. 6b–d). Taken together, these data indicated that the effects of HOXC8 on the proliferation, anchorage-independent cell growth and migration of lung cells were, at least partially, through its regulation of TGFβ1 transcription in NSCLC cells.

### The effects of HOXC8 on the chemoresistance of NSCLC

**Fig. 7:**
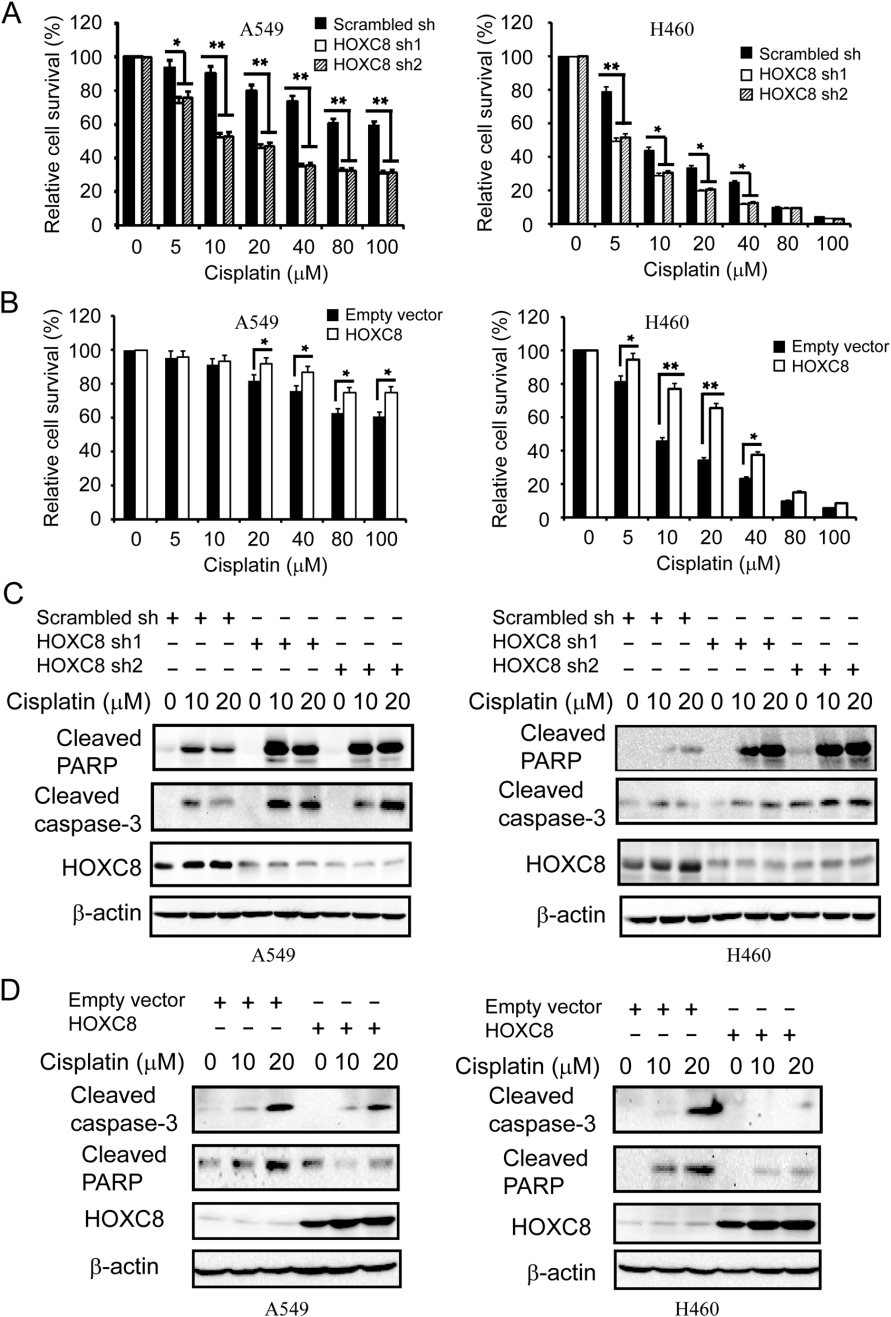
HOXC8 regulates chemosensitivity of NSCLC to cisplatin treatment. **a** A549 or NCI-H460 cells that were lentiviral transduced with scrambled or HOXC8 shRNA were treated with different concentration of cisplatin for 48 h. Cell viability was then measured by MTT assays. Columns, mean; bars, SEM; n = 3; *P < 0.05; **P < 0.01. **b** Cells transduced with empty vectors or HOXC8 expression vectors were treated with different concentration of cisplatin for 48 h. Cell viability was then measured by MTT assays. Columns, mean; bars, SEM; *n* = 3; *P < 0.05; **P < 0.01. **c** Cells transduced with scrambled or HOXC8 shRNA were exposed to the indicated concentration of cisplatin for 48 h. After treatment, cells were lysed and subjected to western blot to detect cleaved caspase-3, cleaved-PARP, HOXC8, and β-actin. **d** Cells transduced with empty vectors or HOXC8 expression vectors were exposed to the indicated concentration of cisplatin for 48 h. after treatment, cell lysates were subjected to Western blot to detect cleaved caspase-3, cleaved-PARP, HOXC8, and β-actin

Currently, most lung cancer patients detected at an advanced stage are ineligible for surgical therapy, and chemotherapy is their first choice of treatment. However, the clinical success of chemotherapy is still limited due to drug resistance^28^. Therefore, efforts to improve the sensitivity of the patients to chemotherapy drugs are highly necessary. Since HOXC8 can promote the proliferation and migration of NSCLC, we further evaluated its role in the chemoresistance of NSCLC. We first examined the effects of HOXC8 on cell viability, in which A549 and NCI-H460 cells were treated with a series dose of cisplatin. We found that HOXC8 knockdown significantly enhanced cisplatin-induced repression on NSCLC viability (Fig. 7a), while HOXC8 ecto-expression attenuated the suppressive effects of cisplatin on both A549 and NCI-H460 cell viability (Fig. 7b). Next, we examined the effects of HOXC8 on cell apoptosis induced by cisplatin treatment. Compared to negative control, cisplatin treatment induced apoptosis, which was evidenced by cleavage of caspase-3 and cleaved PARP in both A549 and NCI-H460 cells. We found that depletion of HOXC8 resulted in higher levels of cleaved caspase-3 and cleaved PARP proteins in cisplatin treated cells (Fig. 7c), which indicated that knockdown of HOXC8 combined with cisplatin treatment enhanced cell apoptosis in NSCLS. Moreover, ecto-expressing HOXC8 decreased the cleavage of caspase-3 and PARP in cisplatin-treated A549 or NCI-H460 cells (Fig. 7d). Taken together, these data indicated that HOXC8 expression could amplify the chemoresistance NSCLC to cisplatin-based chemotherapy and down-regulation of HOXC8 effectively improved the sensitivity of NSCLS to cisplatin treatment.

## Discussion

HOX genes, a highly conserved subgroup of the homeobox superfamily, play crucial roles in embryogenesis and tumorigenesis that share same events such as growth and differentiation, etc^29,30^. The HOX gene expressions that are critical for embryonic development are aberrant in abnormal development and malignancy, indicating that altered expression of HOX genes is important for oncogenesis. Numerous examples of aberrant HOX gene expression have been found in various types of cancer, including breast, prostate, cervical and lung cancer, etc^14,31–33^. In the present study, we demonstrated that HOXC8 was upregulated in NSCLC, and HOXC8 played an important role in proliferation, migration and chemoresistance of NSCLS.

As a member of HOX family, HOXC8 has been found to be deregulated in multiple cancers including breast, colon, cervical, prostate and ovarian cancers^5,8,14,20,34^, and functions as a transcription activator or repressor to regulate a number of genes transcription^13,35–39^. In this study, we investigated for the first time the functions of HOXC8 in NSCLC, and found that HOXC8 was upregulated in human NSCLC clinical specimens compared to normal lung tissues, which was further supported by the meta-analysis using publicly available datasets. Importantly, high expression of HOXC8 was linked to poor relapse-free survival for lung cancer patients (Fig. 1 and Table 1). More importantly, we showed that tumors with upregulated HOXC8 tended to display more aggressive phenotypes (Table 2). Furthermore, gain- and loss-of-function experiments indicated that HOXC8 expression promoted the proliferation, anchorage-independent cell growth and migration of lung cancer cells (Fig. 2). All these data suggested that HOXC8 expression was elevated in NSCLC and played an important role in lung cancer development.

All HOX genes encode transcription factors to regulate the expression of their target genes^40^. On the basis of our observations that HOXC8 ectopically expressing cells undergo EMT-like elongation to exhibit fibroblast like/spindle-shaped cell morphology, we explored whether HOXC8 was involved in regulation of EMT-related genes by ectopically expressing or shRNA depleting HOXC8 expression in A549 or NCI-H460 cells. We found that HOXC8 was probably involved in regulation of TGFβ1, vimentin and E-cadherin expression, which was confirmed by Western blot and qRT-PCR experiments in A549 or NCI-H460 cells (Fig. 3). Although we did not observe that HOXC8 affected the expression of N-cadherin or cadherin-11, we demonstrated that HOXC8 expression markedly decreased E-cadherin expression and increased vimentin and TGFβ1 expression in lung cancer cells, indicating that HOXC8 plays an important role in the EMT process of lung cancer cells.

TGFβ1 is a multifunctional cytokine that plays an important role in a broad range of cellular processes, and numerous studies focus on its roles in signaling pathways^17,41^. However, the molecular mechanisms underlying TGFβ1 regulation are not fully understood. In the present study, we found that HOXC8 silencing led to a decrease in TGFβ1 mRNA and protein levels and HOXC8 ectopic expression increased the mRNA and protein levels of TGFβ1. ChIP analysis revealed that HOXC8 bound to the TGFβ1 promoter on nucleotides −1941 to −1936 upstream of transcription start site, implying that TGFβ1 is a direct target of HOXC8. The promoter luciferase reporter assay further showed that HOXC8 participated in the transcriptional regulation of TGFβ1. In addition, actinomycin-chasing experiments suggested that HOXC8 did not affect TGFβ1 mRNA stability (Fig. 4). All these observations indicated that HOXC8 regulated TGFβ1 expression at its transcriptional level. Furthermore, we found that depletion of HOXC8 decreased the expression of vimentin, which was almost recovered by ecto-expression of TGFβ1 in HOXC8 knockdown cells. Consistently, we further showed that knockdown of HOXC8 significantly inhibited proliferation, anchorage-independent cell growth and migration of lung cancer cells, which can be rescued by ecto-expression of TGFβ1 (Fig. 6). This observation suggested that the effects of HOXC8 on cell growth and migration were, at least partially, due to its induction of TGFβ1 transcription in NSCLC cells.

Regardless of the recent advent of immunotherapy, chemotherapy with platinum compounds still remains one of the most important therapeutic methods to treat NSCLC. Unfortunately, the maximum efficacy of platinum-based chemotherapy could not be achieved clinically due to multiple side effects and emergence of drug resistance phenotype. In the current study, we found that ecto-expression of HOXC8 inhibited cell apoptosis induced by cisplatin treatment, whereas depletion of HOXC8 by shRNA significantly enhanced cisplatin-induced apoptosis in both A549 and NCI-H460 cells (Fig. 7). Since HOXC8 expression is up-regulated in NSCLC patients’ samples compared to normal lung tissues (Fig. 1), the expression of HOXC8 might contribute to poor response to chemotherapy in NSCLC patients. Moreover, the enhanced apoptosis induced by cisplatin in combination with HOXC8 knockdown suggested that the combination of cisplatin with other therapies that modulated HOXC8 could be exploited as a plausible strategy to enhance therapeutic efficacy for NSCLC.

In summary, we demonstrate that HOXC8 is upregulated in NSCLC cells and its upregulation is positively correlated with aggressiveness of NSCLC. We further show that HOXC8 plays an important role in regulation of the proliferation, migration, and chemoresistance in NSCLC. Therefore, our finding suggests that HOXC8 is involved in the tumorigenesis of NSCLC and could be a therapeutic target for human NSCLC disease.

## Materials and methods

### Cells and materials

The human lung cancer cell lines were purchased from the Cell Bank of Shanghai Institute of Cell Biology (Shanghai, China). The cell lines have been authenticated and tested by the Cell Bank. For verification, we performed mycoplasma tests in our laboratory, and the cell behavior and morphology were proved consistent with the descriptions in the Cell Bank. Anti-HOXC8 antibody (15448-1-AP), anti-E-cadherin antibody (20874-1-AP) and Anti-TGFβ1 antibody (20328-1-AP) were from ProteinTech Group (Wuhan, China). Rabbit HOXC8 antibody (HPA028911) for immunohistochemistry was purchased from Sigma (Shanghai, China). Anti-β-actin antibody (sc-1616) was obtained from Santa Cruz Biotechnology (Shanghai, China). Anti-cleaved caspase-3 (#9661), anti-cleaved PARP (#9541) and anti-vimentin (#5741) antibodies were from Cell Signaling Technology (Shanghai, China). Immunohistochemistry kit (#13079) was obtained from Cell Signaling Technology (Shanghai, China). TRIzol RNA extraction reagent was purchased from Sangon Biotech (Shanghai, China). Lipofectamine 2000 and 3000 were purchased from Life Technologies (Shanghai, China). ECL SuperSignal West Femto Maximum Sensitivity Substrate was purchased from Thermo Scientific (Shanghai, China). Cisplatin and all chemical reagents were from Sigma (Shanghai, China).

### Construction of shRNA and gene expression lentiviral vectors

TGFβ1 and HOXC8 shRNA sequences were designed by web-based Invitrogen Block-It program and were subcloned into pLV-shRNA vector (BioSettia, San Diego, CA). TGFβ1 and HOXC8 lentiviral expression vectors were subcloned into pCDH-CMV-MCS-EF1-Puro (System Biosciences, Mountain View, CA). Lentiviruses were prepared as previously described^8^. All oligo sequences of shRNA and PCR primers are included in Supplementary Table S1.

### Western blot

Samples containing equal amounts of protein were separated by SDS–PAGE and electroblotted onto Immobilon-P membranes (Millipore). Western blotting was performed using antibodies as indicated.

### Quantitative reverse transcription-PCR (qRT-PCR)

For RNA expression assays, total RNA was extracted using the Trizol reagent. qRT–PCR was used to analyze the expression of genes. Target gene expression levels were normalized based on β-actin expression levels. The primers that were used for the qRT–PCR analysis are described in Supplementary Table S1.

### Chromatin Immunoprecipitation (ChIP) assay

ChIP assays were carried out as previously described^42^. Briefly, the A549 or NCI-H460 cells were grown to near confluence in 15-cm dishes and fixed in 1% formaldehyde. Sheared chromatin was immunoprecipitated with the anti-HOXC8 antibody overnight at 4 °C. Immune complexes were captured using protein G-agarose, and the formaldehyde cross-links in the eluted complexes were reversed. The DNA was analyzed by PCR or real-time PCR.

### TGFβ1 promoter luciferase assay

TGFβ1 promoter sequence was amplified using genomic DNA isolated from A549 cells. Generated TGFβ1 promoter fragment was subcloned into the pGL4.23 vector (Promega) that contains the firefly luciferase reporter gene. Expression vector encoding Renilla luciferase was included in the transfection for standardization, and a dual luciferase system (Promega) was used to measure luciferase activities according to the protocols of the manufacturer.

### MTT cell proliferation assay

MTT assays were carried out as previously described^13^. Briefly, 5 × 10^3^ cells per well were seeded into 24-well plates and cultured in media containing 10% fetal bovine serum for 1–3 days. Cell viability was tested by adding 3-(4,5-dimethylthiazol-2-yl)-2,5-diphenyltetrazolium bromide (MTT) solution. The color intensity in each plate was read and measured spectrophotometrically using a microplate reader at 560 nm.

### Cell viability assay

The cytotoxic effect of cisplatin and HOXC8 on A549 or NCI-H460 cells were determined by MTT assay. Briefly, 5000 cells/well were seeded in 96-well plates. Next day, cells were treated with different concentration cisplatin for 48 h. Following treatment, cells were incubated with 10 μl MTT (5 mg/ml in PBS) for 2–4 h and then formazan crystals were solubilized by using DMSO. The absorbance was determined by micro plate reader at 560 nm.

### Soft agar colony formation assay

The ability of A549 and NCI-H460 cells for anchorage-independent cell growth was determined by soft agarose assay as previously described^13^. Briefly, cells were subjected to soft agarose assay in 6-well plates and 2 × 10^4^ cells were added into each well which consisted of a bottom base layer (0.6% agarose diluted in DMEM) and top layer (0.3% agarose diluted in DMEM). After 3 weeks, colonies were stained with iodonitrotetrazolium chloride (INT) and counted under a phase-contrast microscope.

### Transwell migration assay

Cell migration was performed, as previously described^8^. Briefly, the undersurface of each Transwell chamber (8 μm pore size; Costar) was coated with 10 μg/ml of Collagen I overnight at 4 °C. Cells were suspended in serum-free medium at a density of 5 × 10^6^ cells/ml, and 100 μl of the cell suspension was added into each Transwell upper chamber. Meanwhile, 10% FBS was added into the lower chamber to serve as a chemo-attractant. After a 4-h migration period, the remaining cells in the upper chamber were removed with cotton swabs, while the cells on the undersurface of the chamber were stained with a crystal violet solution. The number of migratory cells was determined by counting the stained cells in three different fields under a phase-contrast microscope.

### Immunohistochemistry

In total 38 clinical human NSCLC specimens and 18 adjacent non-tumor tissues were obtained from Department of Oncology, Tongji Hospital of Huazhong University of Science and Technology. The study was approved by the Ethical Committee of Huazhong University of Science and Technology. Informed consent was obtained from all the patients before samples were collected, and all samples were collected for research use. Paraffin-embedded tissues were sectioned and used for immunohistochemistry with HOXC8. Sections were also subjected to H&E staining. The scoring of intensity was 0–3 (0 negative; 1 weak; 2 moderate; and 3 strong) and the percentage of positive tumor cells was 0–4 (0 0%; 1 1–25%; 2 26–50%; 3 51–75%, and 4 76–100%). The final scores were obtained by multiplying the intensity and percentage scores. The final multiplied scores of each section were 0–12. Scores of 0–6 were defined as “low expression” and scores of 8–12 as “high expression”.

### Bioinformatics analysis

The expression analysis of HOXC8 were performed using cBioPortal (http://www.cbioportal.org) or Broad GADC Firehose (http://gdac.broadinstitute.org), which is an open-access downloaded bio-database, providing visualization and analyzing tool for large-scale cancer genomics data sets^43^. The Kaplan–Meier analysis was performed using the online Kaplan–Meier Plotter (http://www.kmplot.com) to estimate relapse-free survival curves of lung cancer patients^44^, and the median threshold was used as the cut-off point for the high and low groups of HOXC8 expression.

### Statistical analysis

The data are presented as the means ± SEM. Statistical analyses were performed on data collected from at least three independent experiments. The student’s *t*-test (two-tailed) was used to compare two groups, and differences were considered statistically significant when *P* < 0.05. Statistical analyses were performed with GraphPad Prism with significance levels set at *P < 0.05 and **P < 0.01.

## Electronic supplementary material

supplementary information
