# Supplementary material for: HOXC8 promotes proliferation and migration through transcriptional up-regulation of TGFβ1 in non-small cell lung cancer
Source: Oncogenesis. 2018 Jan 17;7(2):1. doi: 10.1038/s41389-017-0016-4 (PMC5833702; doi:10.1038/s41389-017-0016-4)
Supplement: Supplementary file 1 — supplementary information [file 41389_2017_16_MOESM1_ESM.doc]

**Supplementary information**

Table S1. Oligonucleotides used in real-time PCR, cloning and knockdown studies

| **Primer Name** | **Sequence (5’ to 3’)** |
| --- | --- |
| TGFβ1 promoter cloning |  |
| Forward  reverse | 5’-TTGAGCTCAGGCCATCTCCTGAAGGCACTTGGGAGCCA-3’  5’-AACTCGAGGCCGGGGCCCTCGCTGTCTGGCTGCTCCG-3’ |
| ChIP primers |  |
| Forward-1  Reverse-1  Forward-2  Reverse-2  Forward-3  Reverse-3  Forward-4  Reverse-4  Forward-5  Reverse-5  Forward-6  Reverse-6 | 5’-GGCTTCTGAACTTTGCTGTGAA-3’  5’-CCCTTTGAGTCTTCCACGTAGCT-3’  5’-GGAGCCAGAGCAGGTAATGG-3’  5’-CCTGGGCAACAGAGTGAAACTC-3’  5’-GCCACGTGCCACCACAT-3’  5’-GTGAAACGCCATCTCTATTAAAAAAA-3’  5’-GCCTGGCTGAGATTTCACTTTT-3’  5’-CAGCACCGGAAAGATTTTTTTT-3’  5’-CCTTCTTGCTAAAGCGGTATCC-3’  5’-GTAATTGCTGTGAGGGAAAACAGA-3’  5’-CCACCATCTGATCTACCCTATAATTTG-3’  5’-GCCCCCGTGAAGCTGATA-3’ |
| Knockdown shRNAs |  |
| HOXC8 sh1  HOXC8 sh2  TGFβ1 sh1  TGFβ1 sh2 | 5’-AAAAGCAATATCCCGACTGTAAATCTTGGATCCAAGATTTACAGTCGGGATATTGC-3’  5'-AAAAGCCTCATGTTTCCATGGATGATTGGATCCAATCATCCATGGAAACATGAGGC-3'  5’-AAAAGCAGAGTACACACAGCATATATTGGATCCAATATATGCTGTGTGTACTCTGC-3’  5’-AAAAGCAACAATTCCTGGCGATACCTTGGATCCAAGGTATCGCCAGGAATTGTTGC-3’ |
| Real-time PCR primers |  |
| β-actin forward  β-actin reverse  E-cadherin forward  E-cadherin reverse  N-cadherin forward  N-cadherin reverse  Cadherin-11 forward  Cadherin-11 reverse  Vimentin forward  Vimentin reverse  TGFb1 forward  TGFb1 reverse  TGFb2 forward  TGFb2 reverse  TGFb3 forward  TGFb3 reverse  TGFBR1 forward  TGFBR1 reverse  TGFBR2 forward  TGFBR2 reverse  TGFBR3 forward  TGFBR3 reverse  Twist1 forward  Twist1 reverse  Twist2 forward  Twist2 reverse  Snail1 forward  Snail1 reverse  Snail2 forward  Snail2 reverse  Snail3 forward  Snail3 reverse  ZEB1 forward  ZEB1 reverse  ZEB2 forward  ZEB2 reverse | 5’-TGGATCAGCAAGCAGGAGTATG-3’  5’-GCATTTGCGGTGGACGAT-3’  5’-ACAGCCCCGCCTTATGATT-3’  5’-TCGGAACCGCTTCCTTCA-3’  5’- TGGGAATCCGACGAATGG-3’  5’- GCAGATCGGACCGGATACTG-3’  5’- GCATCCCGCCCATGAGTA-3’  5’- CGCACCCGCAGACTTTG-3’  5’- AATGACCGCTTCGCCAACT-3’  5’- ATCTTATTCTGCTGCTCCAGGAA-3’  5’- GGGAAATTGAGGGCTTTCG-3’  5’- AGTGTGTTATCCCTGCTGTCACA-3’  5’- CGAGAGGAGCGACGAAGAGT-3’  5’- AGGGCGGCATGTCTATTTTG-3’  5’- CTGGCCCTGCTGAACTTTG-3’  5’- AAGGTGGTGCAAGTGGACAGA-3’  5’-CCAGTGTGCTTCGTCTGCAT-3’  5’- CAGTGCGGTTGTGGCAGATA-3’  5’- GCATGAAGGACAACGTGTTGA-3’  5’- TGAGCCAGAAGCTGGGAATT-3’  5’- TGACAGCAGAAACAGAAGAAAGGA-3’  5’- CGGGCCCAATTTAACAGATG-3’  5’- GCGCTGCGGAAGATCATC-3’  5’- GGTCTGAATCTTGCTCAGCTTGT-3’  5’- CCCGGAGACCTCGGTTTT-3’  5’- TCTCGGAGTTGCTGGAAAGG-3’  5’-CCCCAATCGGAAGCCTAACT-3’  5’-GCTGGAAGGTAAACTCTGGATTAGA-3’  5’-CTTGCCCTCACTGCAACAGA-3’  5’-TCTGCAGATGAGCCCTCAGA-3’  5’- TTCCACTGCCACAAACCCTA-3’  5’- AGGCTGGTGTACTCCTTGTC-3’  5’-CACCATCCCCATCACCTCTAA-3’  5’-GCACCCTCAGCTGTGTACAAGT-3’  5’-CGCATTTCCCCCTGCTACT-3’  5’-TGGTCGTAGCCCAGGAATACTG-3’ |
| TGFβ1 expression vector |  |
| XbaI-forward  NotI-flag-reverse | 5’-GCTCTAGAGCCACCATGCCGCCCTCCGGGCTGCG-3’  5’-GGGCGGCCGCTCACTTGTCATCGTCATCCTTGTAATCGCTGCACTTGCAGGAGCGCA-3’ |

**Figure S1**

**
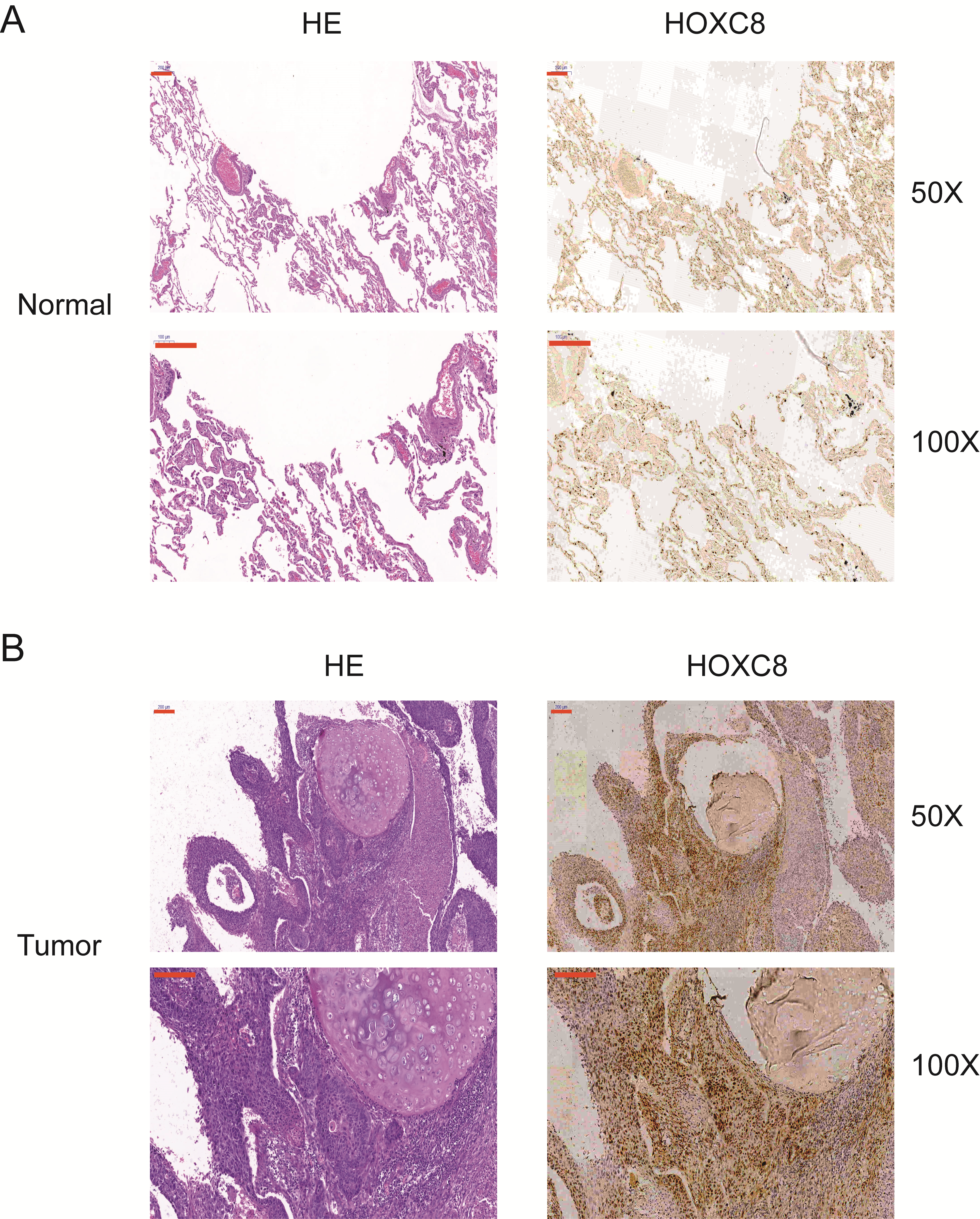
**

**Figure S1. Immunohistochemical expression of HOXC8 in clinical lung cancer specimens and normal lung tissues.** (A) HOXC8 expression was examined by immunohistochemistry in normal lung samples (50×, upper panel; 100×, lower panel). Scale bar, 200μm. (B) HOXC8 expression was determined by immunohistochemistry in lung cancer specimens (50×, upper panel; 100×, lower panel). Scale bar, 200μm.

**Figure S2**


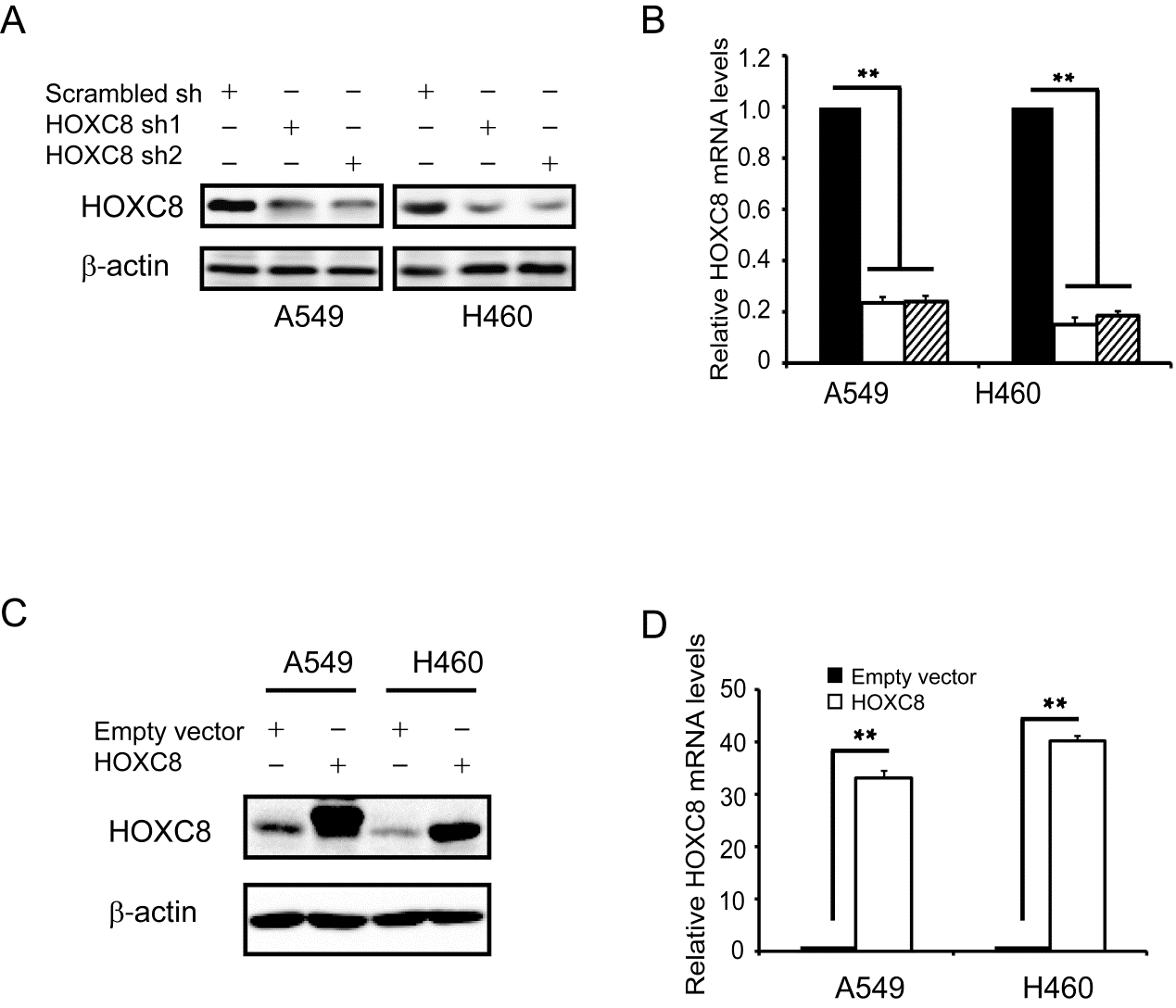


**Figure S2. HOXC8 shRNA silencing or HOXC8 ectopically expressing in NSCLC cells.** (A) A549 or NCI-H460 cells were lentivirally transduced with scrambled or HOXC8 shRNAs. Cell lysates were subjected to Western blot to examine HOXC8 protein levels. β-actin was used as the loading control. (B) A549 or NCI-H460 cells were lentivirally transduced with scrambled or HOXC8 shRNAs. Total RNA was subjected to qRT-PCR to measure the mRNA levels of HOXC8; β-actin mRNA was used as an internal control for standardization. Columns, means; bars, SEM; n = 3: **, *P* < 0.01. (C)A549 or NCI-H460 cells were lentivirally transduced with empty vectors or HOXC8 expression vectors. Cell lysates were subjected to Western blot to examine HOXC8 expression levels. β-actin was used as the loading control. (D) A549 or NCI-H460 cells were lentivirally transduced with empty vectors or HOXC8 expression vectors. Total RNA was subjected to qRT-PCR to measure the mRNA levels of HOXC8; β-actin mRNA was used as an internal control for standardization. Columns, means; bars, SEM; n = 3: **, *P* < 0.01.

**Figure S3**


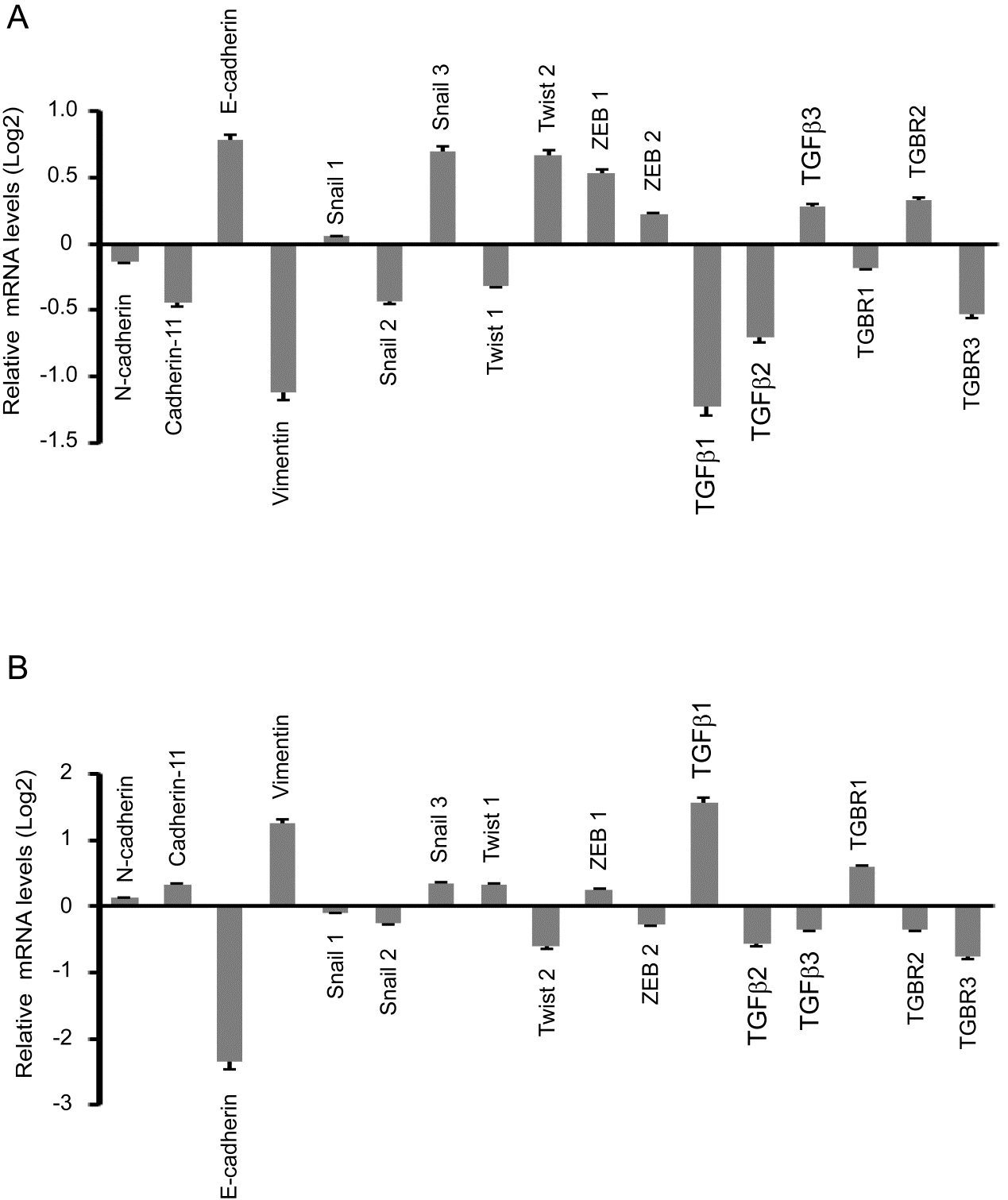


**Figure S3. qRT-PCR to examine the expression of EMT-associated genes.** (A) A549 cells were lentivirally transduced with scrambled or HOXC8 shRNAs. Total RNA was subjected to qRT-PCR to measure the mRNA levels of genes, as indicated; β-actin mRNA was used as an internal control for standardization. Columns, means; bars, SEM; n = 3. (B) A549 cells were lentivirally transduced with empty vectors or vectors encoding HOXC8 protein. Total RNA was subjected to qRT-PCR to measure the mRNA levels of genes, as indicated; β-actin mRNA was used as an internal control for standardization. Columns, means; bars, SEM; n = 3.

**Figure S4**


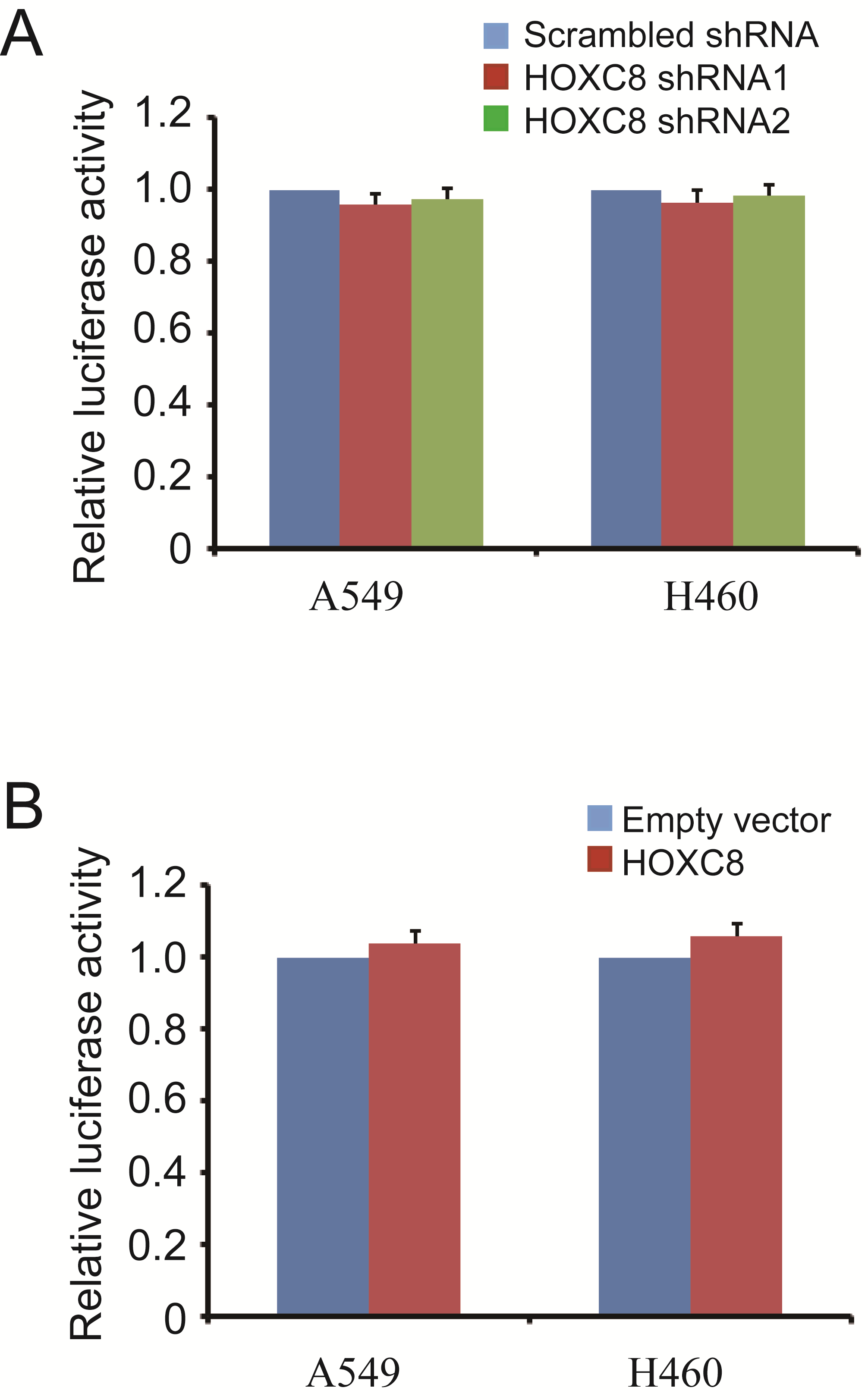


**Figure S4. Mutagenesis of HOXC8 binding site abolished HOXC8 effects on TGFβ1 promoter activities.** Mutant TGFβ1 promoter luciferase reporter vectors were generated by deleting the HOX binding site (nucleotides -1941 ~ -1936 upstream of transcription start site (TSS) of TGFβ1 promoter), and luciferase activities were measured in HOXC8 shRNA knockdown cells (A) or HOXC8 ectopic expression cells (B). Luciferase activity was normalized using Renilla activities. Columns, mean; bars, SEM.
